# Micromechanical Modeling of Fiber-Reinforced Composites with Statistically Equivalent Random Fiber Distribution

**DOI:** 10.3390/ma9080624

**Published:** 2016-07-27

**Authors:** Wenzhi Wang, Yonghui Dai, Chao Zhang, Xiaosheng Gao, Meiying Zhao

**Affiliations:** 1 School of Aeronautics, Northwestern Polytechnic University, Xi’an 710072, China; wangwenzhi@nwpu.edu.cn (W.W.); zhaomeiying@nwpu.edu.cn (M.Z.); 2 Shanghai Aircraft Customer Service Co., Ltd., Shanghai 200241, China; dyhxgd@126.com; 3 Department of Mechanical Engineering, The University of Akron, Akron, OH 44325, USA; xgao@uakron.edu

**Keywords:** fiber-reinforced composites, statistics, random representative volume element, micromechanical, nearest neighbor distance, elastic properties

## Abstract

Modeling the random fiber distribution of a fiber-reinforced composite is of great importance for studying the progressive failure behavior of the material on the micro scale. In this paper, we develop a new algorithm for generating random representative volume elements (RVEs) with statistical equivalent fiber distribution against the actual material microstructure. The realistic statistical data is utilized as inputs of the new method, which is archived through implementation of the probability equations. Extensive statistical analysis is conducted to examine the capability of the proposed method and to compare it with existing methods. It is found that the proposed method presents a good match with experimental results in all aspects including the nearest neighbor distance, nearest neighbor orientation, Ripley’s K function, and the radial distribution function. Finite element analysis is presented to predict the effective elastic properties of a carbon/epoxy composite, to validate the generated random representative volume elements, and to provide insights of the effect of fiber distribution on the elastic properties. The present algorithm is shown to be highly accurate and can be used to generate statistically equivalent RVEs for not only fiber-reinforced composites but also other materials such as foam materials and particle-reinforced composites.

## 1. Introduction

Fiber reinforced composites are known as hierarchical materials with three structural levels: micro-scale, meso-scale and macro-scale. The micro-scale defines the arrangement of fibers in the fiber bundle, the intermediate level (meso-scale) generally relates to the fabric/lamina geometry, and the macro-scale refers to the engineering structural response of the material. In the framework of a multi-scale simulation of composite materials, micro-scale approaches (including both analytical methods and numerical methods) are usually applied to predict the effective stiffness and strength properties of transversely isotropic constitutive properties of composites, serving as theoretical tools for engineering structure design.

The micromechanical analysis is usually performed on a representative volume element (RVE) of the composite. Murthy and Chamis [1] developed a computer code (Integrated Composite Analyzer—ICAN) to calculate the stiffness and strength properties of a composite using a geometry, where one cell contains pure resin and another cell contains fiber. Aboudi [2] developed the so-called “method of cells” to model the continuously reinforced, unidirectional fibrous composite as a doubly periodic array of fibers embedded in a matrix phase. The unit cell consists of a single fiber subcell surrounded by three matrix subcells. The analysis of the RVE consists of the imposition of displacement and traction continuity conditions at the interfaces of the elements as well as the interfaces between neighboring elements, in conjunction with equilibrium conditions. Herakovich [3] described a concentric cylinder model (CCM) that assumes a cylindrical fiber surrounded by a tube shaped interface and matrix. This model achieves good prediction of normal engineering constants, but is unable to predict the transverse shear modulus. Goldberg et al. [4] developed a micromechanical model, where the composite unit cell is divided into a number of slices. Micromechanics equations are then developed for each slice, with laminate theory applied to determine the elastic properties, effective stresses and effective inelastic strains for the unit cell. Sun and Vaidya [5] described a method based on the finite element analysis of an RVE to predict the effective mechanical properties of a unidirectional fiber composite and discussed the importance of the boundary conditions imposed on the RVE. More recently, Sonon and Massart [6] presented a framework for the computational homogenization of the mechanical properties of textile reinforced composites.

Most of the existing methods regard the micro-scale geometry as a periodic structure, assuming a deterministic and ordered distribution of fibers. However, the realistic distribution of fibers has been known to be non-uniform and randomly distributed. Therefore, methods based on periodic fiber distributions cannot give accurate predictions of the effective properties of the composite, especially on the elastic-plastic behavior under transverse loading conditions, due to the inadequate modeling of the resin-rich region and the fiber-aggregate region [7].

Extensive studies on the effect of non-uniform fiber distribution on the overall composite behaviors are reported in [8,9,10]. Sun et al. [11] built random and periodic unit cell models for an E-glass particle reinforced composite and studied the lower bound and upper bound of Yong’s modulus under iso-displacement loading and iso-stress loading, respectively. By comparing with experimental data, the random unit cell model shows better prediction of overall bounds for elastic properties. Trias et al. [12] demonstrated that random models must be considered for the simulation of local phenomena, as the use of periodic models leads to underestimation of matrix cracking and damage initiation. Hojo et al. [13] studied the effect of local fiber array irregularities (e.g., interfiber distance and fiber alignment angle) on the microscopic interfacial normal stress states for thermally and transversely loaded unidirectional carbon fiber (CF)/epoxy composites, where the 2D scanning electron micrograph was used as the basis for generating the finite element models. Zhang and Yan [14] found that the rectangular and hexagonal periodic models show inconsistency in predicting the transverse normal and shear modulus, and both rectangular and hexagonal periodic models show insufficient accuracy compared with the random model.

**Figure 1:**
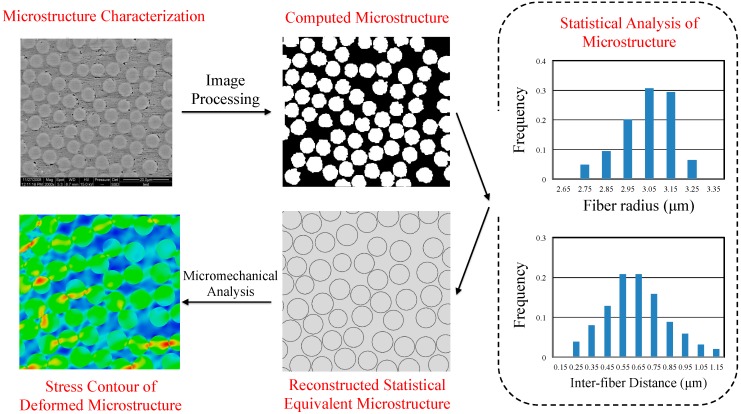
Flowchart for micromechanical modeling of random representative volume element. The scanning electron microscope (SEM) image is reproduced from [15].

The general approach for modeling the non-uniform spatial arrangement of fibers includes characterization of the microstructure, statistical analysis of the microstructure, reconstruction of the random RVE model and micromechanical modeling of the RVE. Figure 1 illustrates the modeling flowchart. Firstly, the realistic microstructure with fiber distribution is characterized using, for example, a scanning electron microscope (SEM). Digital image analysis is then carried out on the original image to identify the fibers based on a color threshold algorithm. From this image, the information such as the distribution of fiber radius and the distances between neighboring fibers can be extracted. The obtained statistical parameters are utilized to reconstruct a statistically equivalent RVE based on certain numerical algorithms. Finally, the reconstructed RVE is meshed to create a finite element model, which is used to predict the effective mechanical performance.

To generate a statistically equivalent RVE, the hard-core model (also called the random sequential adsorption model) has been widely used [16,17,18]. This method creates a set of randomly distributed points inside a square region, with the constraint that no pair of points may be closer than a certain minimum distance. Yang et al. [19] simulated the non-uniform microstructure of a ceramic matrix composite using the hard-core model and validated the statistical equivalency of the reconstructed random model against the realistic fiber distribution. However, the hard-core model does not permit the fiber volume fraction to be greater than ~54.7% due to the presence of the so-called “jamming limit” [20]. To overcome the jamming configuration of the hard-core model, a shaking process can be included to increase the number of inclusions through giving each fiber a small random displacement independent of its neighbor fibers. A detailed description of various algorithms for shaking or vibration of dense packing can be found in Barker [21]. Wongsto and Li [22], Zhang and Yan [14] and Wang et al. [23] obtained non-uniform fiber distributions through disturbing the initially periodical hexagonal/square fiber arrangement. Melro et al. [24] developed a three-step procedure for generation of random fiber distribution, which includes the hard-core model (initial generation of fibers), stirring the fibers (assigning a small random disturbance to each initial generated fibers) and fibers in the outskirts (further generation of fibers in the matrix rich regions produced during the second step). Melro et al.’s method, i.e., the random microstructure generation (RMG) algorithm, shows the capability of achieving a fiber volume ratio of 65%. Romanov et al. [9] conducted an extensive statistical analysis on the validity the RMG model against the experimental data and found a good correlation between the statistical parameters for the real and simulated fiber arrangements. Vaughan and McCarthy [25] developed a combined experimental-numerical approach, the nearest neighbor algorithm (NNA), that generated fiber distribution with the same geometric features as the experimental samples determined using statistical analysis, reproducing both the short and long range interaction of fibers. The NNA model is fast in computational time, but, statistically, it produces a larger frequency of small inter-fiber distances and a lower frequency of large inter-fiber distances. Yang et al. [26] proposed a random sequential expansion (RSE) algorithm based on a hard-core model, which is able to generate random distributions for various fiber volume fractions through adjusting the inter-fiber distance parameters. However, the inter-fiber distances of the RSE model are limited within the nearest neighbor distance, which could result in fiber aggregation at the center area and matrix rich region at the corners. Liu and Ghosha [27] proposed a set of criteria for examining the geometrical equivalence of two microstructures based on the probability functions, convergence of microstructure size and equality of fiber radius distribution.

In this paper, a modified NNA method is proposed to generate the statistically equivalent random RVE, which overcomes the problem of low nearest fiber distribution produced by the original NNA method. Section 2 provides the details of this new method. Section 3 presents the statistical analysis to validate the new method and compares it with existing methods. Finally, in Section 4, the proposed method is applied to predict the elastic constants of a carbon/epoxy composite.

## 2. Algorithm Development

**Figure 2:**
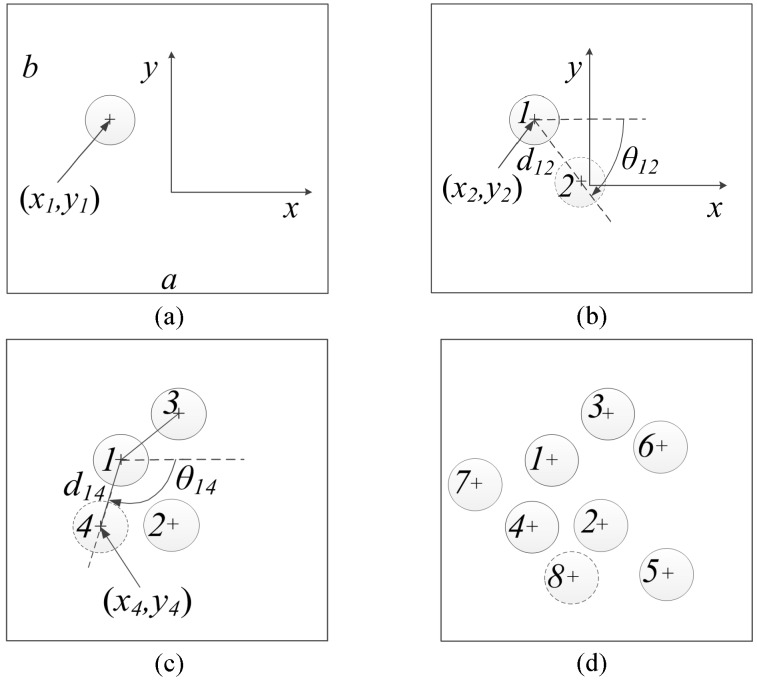
Schematic illustration of the modified nearest neighbor algorithm (NNA) algorithm. (**a**) Step 1: generation of the first fiber (fiber #1) in the central area of the window; (**b**) step 2–4: generation of the fiber #2 with inter-fiber distance *d*_12_ and the orientation angle *θ*_12_ satisfying the statistical distribution; (**c**) step 5–6: generation of more fibers around fiber #1; (**d**) step 7–8: repeating of the previous steps followed by switching the reference fiber.

Previous research [9,27] has shown that RVEs having similar statistical distribution (also known as statistical equivalency) of inter-fiber distances, orientation angles and fiber diameters with those of experimental characterization perform better in predicting the effective mechanical properties. In order to generate a statistically equivalent RVE for a composite microstructure of high fiber volume ratio, a new algorithm is developed on the basis of the NNA method. In the NNA model, the generated RVE generally presents a larger frequency of the smallest inter-fiber distance than that of the predefined parameters. Thus, probability equations are introduced in the present method to control the nearest neighbor distribution and to match the statistical equivalency of the microstructure. The procedure of the proposed algorithm is described below and is also illustrated in Figure 2.

1. Consider a rectangular objective window with length *a* and width *b*, a random fiber is first created in the central area of the window with the coordinates of the center (*x*_1_, *y*_1_), as shown in Figure 2a. The diameter of fibers can be identical or variable, and, in this study, the values are drawn from the experimentally measured diameter distribution. The RVE size must be large enough to be representative in predicting the mechanical properties of the composite material. As from the study of Trias et al. [12], a minimum length and width of 50 times of the fiber radius is recommended.
2. Following the realistic nearest neighbor distribution, a nearest neighbor distance is assigned to the newly generated fiber (for example, fiber #1 for the first cycle).
3. A new fiber (fiber #2) is then generated around the current reference fiber (fiber #1). The position of the new fiber is calculated based on the inter-fiber distance *d_12_* and the orientation angle *θ*_12_ (see Figure 2b), which are determined based on the probability equation and the distribution of nearest fiber distance and orientation angle.
4. In the original NNA method, the newly generated fiber (fiber #2) is checked and if the inter-fiber distance between it and the reference fiber is less than the nearest neighbor distance of the reference fiber, it will be regenerated. This rule could result in the increase of the inter-fiber distance and a relatively low frequency of small inter-fiber distance distribution. To overcome this issue, a random number *P* with the value between 0 and 1 is generated and is associated with a probability selection rule. If *P* is less than a predefined threshold value *P*_0_ (0 < *P*_0_ < 0.3), the newly generated fiber will be kept even though it does not satisfy the nearest neighbor distance. *P*_0_ is taken as 0.15 in this work.
5. Steps 2–4 are repeated to generate more fibers around the current reference fiber, until the maximum iteration numbers (usually between 3 and 5) are reached. The inter-fiber distances between each newly generated fiber and all the existing fibers need to be examined separately. For example, in Figure 2c, the inter-fiber distance between fiber #1 and fiber #4 should be larger than the nearest neighbor distance of fiber #1. However, the probability selection rule of step 4 always applies.
6. Followed by the generation of each new fiber, the frequency of the nearest neighbor distance of fibers needs to be updated. The equation is expressed as
(1) $$L_{i}=\frac{L_{i}^{0}N-N_{i}}{N-\sum N_{i}},$$
  where *L_i_* is the updated frequency of nearest neighbor distance for the *i*th cycle (each cycle corresponds to a different fiber serving as the reference fiber), $L_{i}^{0}$ is the initial frequency of nearest neighbor distance for the *i*th cycle, *N* represents the estimated total number of fibers, and *N_i_* represents the fibers already generated during the *i*th cycle.
7. The current cycle ends when there is no more new fiber generation around the current reference fiber. The reference fiber is then switched to a different fiber based on the fiber number, e.g., fiber #2 will be the reference fiber of the second cycle.
8. The above process is repeated until the requested fiber volume ratio or maximum number of cycles is reached.

**Figure 3:**
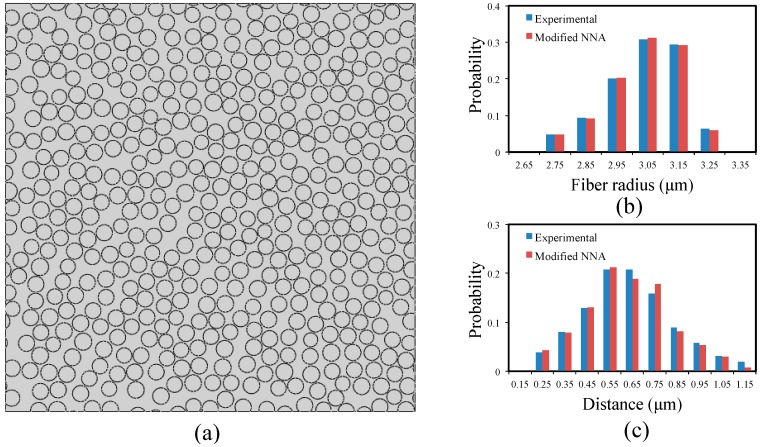
(**a**) Example of generated representative volume element (RVE) of fiber volume ration 57%; (**b**) distribution of fiber radius; and (**c**) distribution of nearest neighbor distances.

From the above procedures we can see that the overall flowchart is similar to the existing methods (RSA, RSE and NNA), while the introduction of the probability equation (step 4) and the frequency updating equation (step 6) enables a closer representation of the realistic fiber distribution. The proposed algorithm is implemented in MATLAB and utilized to produce a representative RVE for the carbon/epoxy composite (fiber volume ratio 57%) shown in Figure 1. Figure 3a shows an example of generated random RVE, where the width and length of the RVE are 50 times that of the average fiber radius. Compared with the SEM image shown in Figure 1, the RVE possesses very similar microstructure features as in the original micrograph, such as resin rich regions, fiber aggregation zone and “lines” of fibers. Figure 3b,c compare the generated fiber radius and inter-fiber distance distributions using the modified NNA method with the experimental measurements. It can be seen that the proposed algorithm results in very good representation of the realistic fiber distribution. The radius of fibers ranges from 2.75 to 3.25 μm. The nearest inter-fiber distance is about 0.25 μm, which has a frequency of 3.9%.

The average computation time is about 76 s on a 3.4 GHz, 8 GB RAM personal computer for an RVE with the width and length of 50 times the average fiber radius. This is relatively longer than the existing methods (RSE, RSA and NNA) due to the introduction of additional probability equations. In addition, the proposed method requires extracting parameters from the experimental characterization results of the SEM images for the modeled fiber volume ratio or obtaining the dependency of fiber arrangements on fiber volume ratio for the studied material. However, compared with the time needed to run the finite element analysis, the time spent on generating the random RVE is negligible. More importantly, the significantly enhanced equivalency in capturing the realistic fiber distribution provides a promising path on studying the effect of random fiber distribution on the elastic and strength properties of composites.

## 3. Statistical Characterization

To verify the validity of the presented methodology, statistical analysis is conducted to quantitatively characterize the fiber distribution of generated RVE. Different statistical functions can be used to describe the spatial distribution of fibers. Melro et al. [24] and Romanov et al. [9] presented the details of the statistical spatial descriptors for random fiber arrangement, including Voronoi polygon areas, neighboring fiber distances, nearest neighbor distances, nearest neighbor orientation, Ripley’s K function, radial distribution function, etc. In this section, the statistical equivalency of the RVE generated using the proposed method is studied in four aspects: nearest neighbor distances, nearest neighbor orientation, Ripley’s K Equation (second-order intensity function) and radial distribution function. To highlight the improvement of the current method, a comparison study with existing methods is also presented.

### 3.1. Nearest Neighbor Distance

**Figure 4:**
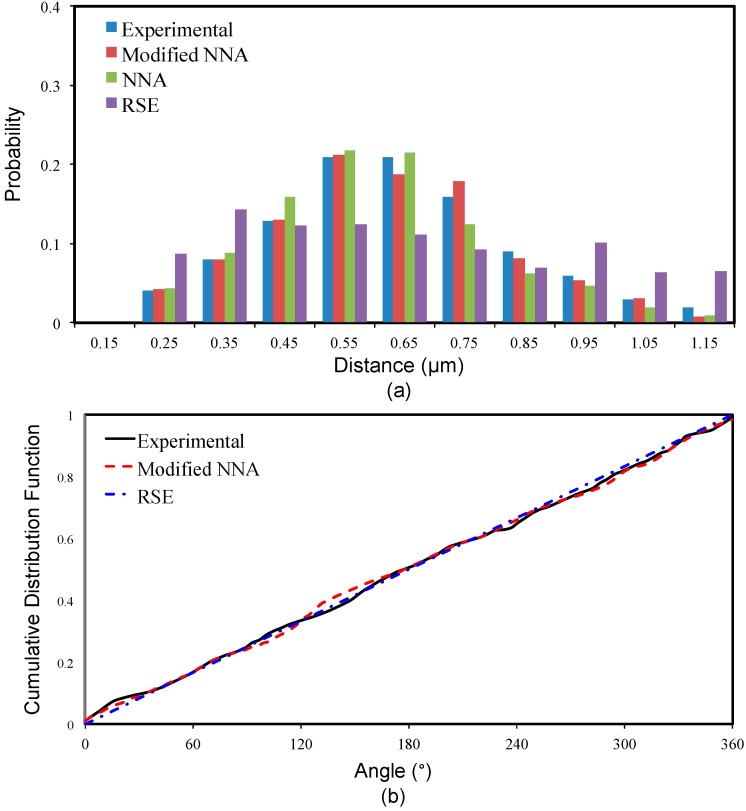
Comparison of experimental measured and computational modeled (**a**) probability density function of nearest neighbor distances and (**b**) cumulative distribution function of nearest neighbor orientation.

The nearest neighbor distance of a fiber is defined as the minimum value of inter-fiber distances between the reference fiber and neighbor fibers. Hojo et al. [13] found that the nearest neighbor distance has a significant effect on the stresses developed in the fiber/matrix interface, which, in turn, has significant impact on the consequential failure behavior of the composite material. A concentration of nearest neighbor distance (or fiber aggregation) is more likely to occur at a high fiber volume ratio, where a larger amount of fibers is present in an RVE of fixed dimension. The distribution of nearest neighbor distance is generally evaluated using the probability density function (PDF), which defines the frequency of a random distance. The PDF of nearest neighbor distance is sensitive to the fiber aggregation and provides information of the interaction between inter-fiber distances of fibers. In the statistical analysis of microstructure for fiber-reinforced composites, PDF is usually used to interpret the feature of fiber arrangement. The PDF plot can help identify the presence of fiber aggregation, which generally shows as a sharp peak in the PDF plot at a certain distance value. Sometimes, the second nearest fiber distance distribution will be introduced to further examine the presence of fiber aggregation [25]. Figure 4a compares the nearest neighbor distance distribution of the experimental results and the random RVEs generated by different methods (NNA, RSE and modified NNA). Basically, the experimental results follow a Gaussian distribution. The modeling results for each method are obtained through averaging the statistical information of 20 random RVEs. It can be seen from Figure 4a that the current method performs the best in modeling the distribution of nearest neighbor distance. The NNA method has a relatively higher probability density for distances lower than the average nearest neighbor distance but a lower probability density for distances higher than average nearest neighbor distance. The RSE method has an almost uniform density for all distances showing an obvious discrepancy from experimental results.

### 3.2. Nearest Neighbor Orientation

The nearest neighbor orientation is the distribution of the orientation of the undirected line connecting the centers of the reference fiber with its nearest neighbor, which is generally described using cumulative distribution function (CDF). It is calculated as the rotation angle from the horizontal axis (*x*-axis of Figure 2) clockwise to the line connecting the reference fiber and its nearest neighbor. The CDF takes into consideration all fibers in a RVE, and it is close to a straight diagonal line for a completely spatial random (CSR) pattern of fibers corresponding to an equal probability for the occurring of all orientations. If the distribution of fibers shows a concentration at a certain angle (for example, a periodic distribution), the CDF curve will be a stair-shape showing an obvious discrepancy from the diagonal line. Figure 4b compares the experimental and modeling CDF curves, where we can see the RSE curve is almost a diagonal line due to its usage of uniform random distribution. The experimental curve and modified NNA curve both show a slightly vibration but still follow the tendency of diagonal line. The curve for the NNA method is not shown here for visibility, as it is very similar to the modified NNA curve. Overall, the present method and other existing methods are all able to capture well the orientation randomness of fiber distribution.

### 3.3. Ripley’s K Function

The Ripley’s K function, also known as the second-order intensity function, has been known as one the most informative descriptors for spatial patterns [18]. It can be used to distinguish distribution behavior and its interaction with distances from the fiber center. The Ripley’s K function counts the ratio of the number of fibers within a radial distance *h* for an arbitrary fiber against the number of fibers within a unit area. It is estimated by [28]:

(2) $$K(h)=\frac{A}{N^{2}}\sum_{i=1}^{N}w_{i}^{-1}I_{i}(h),$$

where *A* is the area of the objective window, *N* is the total number of fibers in the window, *I_i_*(*h*) is the number of fibers lying within radial distance *h* of a given fiber and *w_i_* is the ratio of the circumference contained within the window to the whole circumference of the circle. For a CSR pattern, the above equation can be simplified as

(3) Kcsr(h)=πh2NANA=πh2.

**Figure 5:**
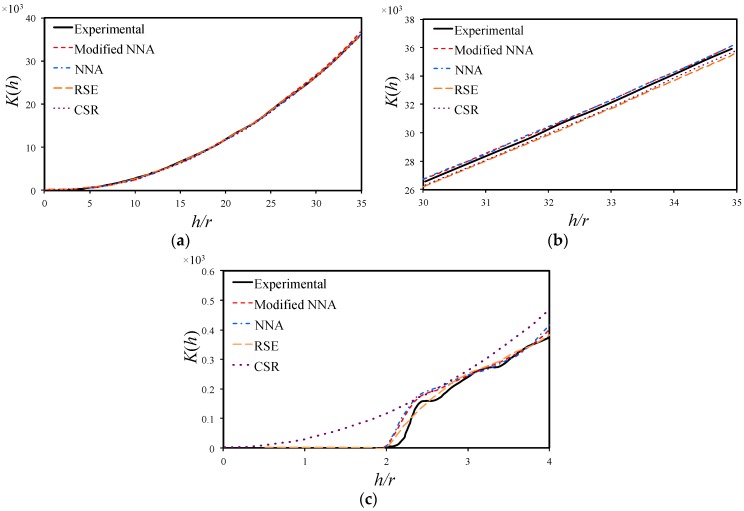
Comparison of experimental measured and computational modeled Ripley’s K function (**a**) 0 ≤ *h/r* ≤ 35; (**b**) 30 ≤ *h/r* ≤ 35; and (**c**) 0 ≤ *h/r* ≤ 4.

A comparison of the *K*(*h*) and *K_csr_*(*h*) curves can help in understanding the fiber distribution behavior. As discussed by Yang et al. [26] and Melro et al. [24], it indicates that the distribution shows some degree of regularity if the curve of *K*(*h*) lies below the *K_csr_*(*h*) curve, while fiber aggregation presents when the curve of *K*(*h*) goes above the *K_csr_*(*h*) curve. Similar to the cumulative distribution function, the *K*(*h*) curve will show a stair-shape when the fibers have a periodical distribution (square distribution or hexagonal distribution). Figure 5a shows the Ripley’s K function for the numerically generated microstructures using the modified NNA, NNA, RSE and CSR methods, together with the experimental curve. The abscissa is normalized as *h/r*, where *r* is taken as the average fiber radius of 3.05 μm. All curves in Figure 5a show almost the same increasing tendency of *K*(*h*) against *h* for the range of *h/r* from 0 to 35.

Figure 5b,c present the zoomed plots of Figure 5a for the range of *h/r* from 30 to 35 and from 0 to 4, respectively. As from Figure 5b, all curves of *K*(*h*) increase linearly with respect to *h/r*, and the *K*(*h*) of NNA and modified NNA are relatively higher than that of the CSR method. As in Figure 5c, the experimental curve and numerical curve of NNA, RSE and modified NNA methods show an irregular shape indicating the presence of a certain regularity of fiber distribution at low distance range (*h/r* < 4). In the mean time, the *K*(*h*) curve of CSR is obviously different from the others, showing a continuously increasing tendency starting from 0. These are consistent with the results reported by Vaughan and McCarthy [25].

For better comparison of *K*(*h*) from different methods, the equation below is introduced to enhance the understanding of fiber distribution

(4) $$L(h)=\sqrt{\frac{K(h)}{\pi }}-h.$$

**Figure 6:**
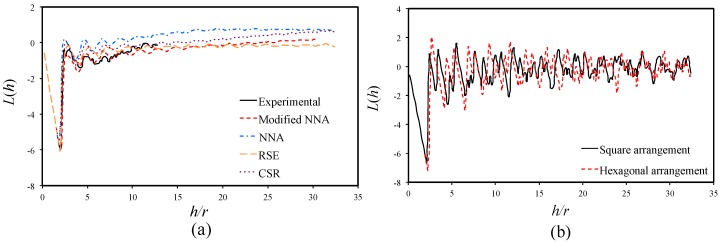
(**a**) *L(h)* for experimental characterized and numerical generated RVE; and (**b**) *L(h)* for periodic RVE of square arrangement and hexagonal arrangement.

As from Equation (4), *L*(*h*) of CSR pattern will be a constant 0. Figure 6a shows the *L*(*h*) plots for the experimental result and the results from the generated RVE using different methods. Due to the limitation of the experimental data (size of the SEM image), the *h/r* of the experimental curve is limited to the range of 0 to 15. The positive peaks of the curves indicate the presence of fiber aggregation with respect to the CSR pattern, while the negative peaks correspond to a regular distribution. As from Figure 6a, all experimental and numerical curves show an obvious oscillation in the range of *h/r* from 3 to 10, and gradually stabilize as *h/r* increases. The first negative peak corresponds to the smallest fiber radius, below which the material is purely uniform. With the increase of radial distance, the fiber aggregation and resin rich zone increase corresponding to the oscillation of the curve.

Figure 6b gives an example of *L*(*h*) vs. *h/r* curves for RVEs of square arrangement and hexagonal arrangement, where we can see regular positive and negative peaks along the whole distance span. The amplitude of the curve oscillation decreases and the frequency of the curve oscillation increases with the increase of *h*. This also indicates an increase of regularity due to the radial distance *h*.

It is also noticed in Figure 6a that the modified NNA method matches the experimental results best. According to the results of Figure 5 and Figure 6, Equation (4) is more feasible in elaborating the capability of the current algorithm in producing statistically equivalent RVE.

### 3.4. Radial Distribution Function

**Figure 7:**
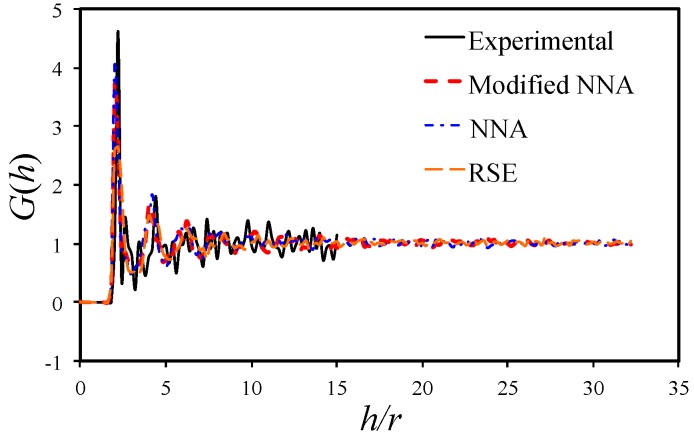
Radial distribution function for experimental characterized and numerical generated RVE.

The radial distribution function, also known as the pair distribution function, describes how the average fiber density varies against the distance from a given fiber. It is determined as the ratio of the number of fibers lying within an annular region of inner radius *h* and outer radius *h* + d*h*, over the average number of fibers within a unit area. Mathematically, it can be expressed as [29]

(5) $$G(h)=\frac{1}{2\pi N_{h}^{a}\cdot dh}\cdot \frac{1}{N}\sum_{i=1}^{N}n_{i}(h)=\frac{dK(h)}{2\pi h\cdot dh},$$

where d*K*(*h*) and *n_i_*(*h*) are defined as the average number and the total number of fibers lying within an annulus of inner radius, *h*, and outer radius, *h* + d*h*, respectively. *N_a_* is the number of fibers per unit area. While Ripley’s K equation is generally used to distinguish the distribution behavior, the radial distribution is utilized to study the distance density. Figure 7 compares the radial distribution function for numerical generated microstructures and the experimental result. A sharp peak is observed at *h/r* = 2.2, corresponding to an inter-fiber distance of 0.2*r* (0.61 μm), which coincides with the peak of nearest neighbor distance distribution (Figure 3c). As can be seen from this figure, the function exhibits some fluctuations for the medium range (i.e., 3 < *h/r* < 15), typical of a high volume fraction composite [25]. It then approaches unity, with the distance *h* becoming large enough to be representative of a region of perfect randomness. Romanov et al. [9] found that the realistic radial distribution for fiber arrangement always saturates at unity for a sufficient large distance *h*. The capability to match this behavior is an important measure for statistically equivalency.

As from Figure 7, the curves for the numerical generated microstructures converge to unity at a relatively low distance range (*h/r* < 15), while the experimental result still oscillates at *h/r* = 15. The curve obtained with the RSE method converges to unity very fast, while the curves obtained with the NNA method and the modified NNA method match well with experimental data in the initial stage (*h/r* < 5) before converging to unity in the *h/r* range between 10 and 15. The *G*(*h*) of the CSR pattern is always one and is not plotted in Figure 7.

## 4. Prediction of Elastic Properties

One of the objectives of generating statistically equivalent RVEs is to predict the effective mechanical properties of the composite material from the properties of its constituents. In the following example, random RVEs are generated using the modified NNA method to predict the elastic properties of a carbon fiber reinforced epoxy composite T300/914C.

Finite element models are generated in ABAQUS [30] (Version 6.11, Dassault Systemes Corp., Providence, RI, USA) with plane strain quadrilateral elements (CPE4) and plain strain triangular elements (CPE3) for two-dimensional (2D) models, and hexahedral elements (C3D8R) and tetrahedral elements (C3D6) for three-dimensional (3D) models. The 3D model is utilized to predict the longitudinal normal modulus *E*_11_, shear modulus *G*_12_/*G*_13_ and Poisson’s ratio *v*_12_/*v*_13_ while the 2D model is used for predicting the transverse normal modulus *E*_22_/*E*_33_, shear modulus *G*_23_ and Poisson’s ratio *v*_23_.

**Figure 8:**
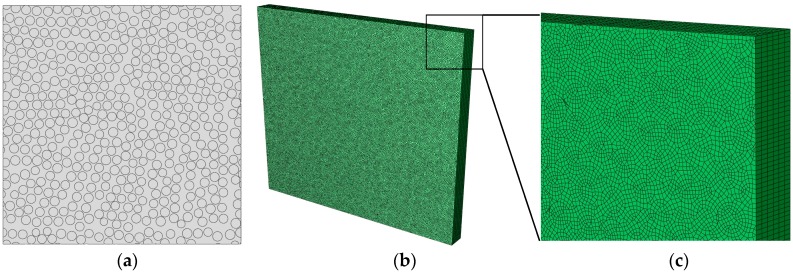
(**a**) Random RVE for composite with fiber volume ratio 60%; (**b**) and (**c**) 3D finite element mesh for the microstructure of (**a**).

The previous analysis is conducted based on the experimental results of SEM image in Figure 1, which has a fiber volume ratio of 57%. With the purpose of comparing with the experimental results of a carbon fiber reinforced epoxy composite T300/914C of fiber volume ratio 60% [31], we assume the T300/914C composite has the same fiber distribution as the SEM image of Figure 1 with the number of fibers scaled-up proportionally (60/57 = 1.05) against the fiber radius distribution and inter-fiber distribution of Figure 3b,c. Figure 8a shows a generated RVE using the modified NNA method described in Section 2. Each RVE contains about 500 fibers. A Python script is written to read the circular center and radius information to generate the geometry. Figure 8b,c show the 3D finite element mesh of the random RVE shown in Figure 8a. A total of eight RVEs with different fiber distributions are generated with an average element size of about 0.8 μm, which results in a total of about 50,000 elements for the 2D model, and about 550,000 elements for the 3D model with 11 elements through the thickness direction. For simplicity, perfect interface is imposed, meaning no sliding or failure between fibers and matrix.

The fiber and the matrix are considered to be transverse isotropic and isotropic, respectively. Their mechanical properties are obtained from [31] and listed in Table 1. When modeling composites using an RVE, it is important to include the appropriate periodic fiber distribution and apply appropriate boundary conditions [32]. Sun and Vaidya [5] described how to perform finite element analysis of an RVE to predict the effective mechanical properties of a unidirectional fiber composite. They discussed methods for properly imposing boundary conditions for a variety of loading cases. The application of periodic boundary conditions on the numerically generated random RVEs follows the same mathematical equations as suggested by Sun and Vaidya [5]. In ABAQUS, the periodical boundary conditions are applied through “Equation Constraints” [30]. The RVEs are deformed through applying a displacement on a rigid reference node that is kinematically coupled with the RVE surfaces in the loading direction so that the displacements for all boundary nodes are consistent with those of the equivalent nodes on the opposite surfaces.

To determine the appropriate elastic properties in all coordinate directions, four load cases have to be analyzed, axial tension, transverse tension, longitudinal shear and transverse shear, using the mechanical properties of the fiber and matrix provided in Table 1. The effective elastic properties are estimated using a volume average approach, with equations shown below [12]:

(6) $$\text{Longitudinal Tensile}:E_{11}=\sum_{i=1}^{N}\frac{\sigma_{11}^{i}V_{i}}{\varepsilon_{11}^{i}V_{i}}\;\;v_{12}=\sum_{i=1}^{N}\frac{\varepsilon_{22}^{i}V_{i}}{\varepsilon_{11}^{i}V_{i}}\;,$$

(7) $$\text{Transverse Tensile}:E_{22}=\sum_{i=1}^{N}\frac{\sigma_{22}^{i}V_{i}}{\varepsilon_{22}^{i}V_{i}}\;\;v_{23}=\sum_{i=1}^{N}\frac{\varepsilon_{33}^{i}V_{i}}{\varepsilon_{22}^{i}V_{i}}\;,$$

(8) $$\text{Longitudinal Shear}:G_{12}=\sum_{i=1}^{N}\frac{\tau_{12}^{i}V_{i}}{\gamma_{12}^{i}V_{i}}\;,$$

(9) $$\text{Transverse Shear}:G_{23}=\sum_{i=1}^{N}\frac{\tau_{23}^{i}V_{i}}{\gamma_{23}^{i}V_{i}}\;,$$

where *N* represents the number of elements in the RVE model, *V_i_* is the volume of the *i*th element, $\sigma_{kl}^{i}$ , $\tau_{kl}^{i}$ , $\varepsilon_{kl}^{i}$ and $\gamma_{kl}^{i}$ (*k*, *l* = 1, 2 and 3) corresponds to the normal stress, shear stress, normal strain and shear strain values in the appropriate directions obtained from the finite element simulation.

The effective elastic properties of the eight random RVEs are computed using Equations (6)–(9). In addition, the average values and standard deviations of the eight sets of computed elastic properties are calculated. The numerical values, together with the experimental results from [31], are summarized in Table 2. Overall, the current model predicts the elastic properties in a good manner, especially for the longitudinal properties. The discrepancy on the transverse properties is encountered by many researchers [20,21,22,33]. The reason for this is because interphases, voids and other internal defects existing in the actual material but not considered in the numerical model. On the other hand, the standard deviation values of the predicted elastic properties are very small, indicating that the randomness of fiber radius and fiber arrangement has little effect on the effective elastic properties of the composite. However, as shown in [13], the randomness of fiber radius and fiber arrangement does have a significant effect on the local failure process and the effective strength properties of the composite. An extensive study of the effect of random fiber arrangement on the interfacial failure and effective strength properties is ongoing and will be presented in a future publication.

## 5. Conclusions

A new algorithm, named “modified NNA”, is developed in this work for generating statistically equivalent RVEs of fiber-reinforced composites. The modification to the NNA method is accomplished by implementing experimentally measured fiber radius and inter-fiber distance distributions and introducing probability equations into the algorithm. The proposed method has demonstrated a significant advantage in capturing the realistic fiber distribution compared with existing methods. Extensive statistical analysis is conducted to elaborate the accuracy of the current method in generating the statistically equivalent RVE. It is found that the proposed method presents a good match with experimental results in all aspects including the nearest neighbor distance, nearest neighbor orientation, Ripley’s K function and radial distribution function. The numerical analysis also showed that the CSR pattern cannot represent the realistic fiber distribution. Finite element models are developed with the random RVEs generated using the current method. The results indicate that the randomness of fiber distribution has little effect on the effective elastic properties, resulting in very small standard derivation values for all predicted elastic constants. Further study of the randomness of fiber distribution on the effective strength properties of the composite is ongoing. Although this paper focuses on fiber-reinforced composites, the developed algorithm is fully applicable for generating micromechanical models for other materials such as foam materials and particle-reinforced composites.

## Figures and Tables

**Table 1:** Elastic properties of T300 carbon fiber and 914C epoxy resin [31].

Properties	Carbon Fiber T300	Epoxy Resin 914C
Longitudinal Young’s Modulus *E*_1_ (GPa)	230	4
Transverse Young’s Modulus *E*_2_ (GPa)	15	4
Longitudinal Shear Modulus *G*_12_ (GPa)	15	1.481
Transverse Shear Modulus *G*_23_ (GPa)	7	1.481
Major Poisson’s Ratio *ν*_12_	0.2	0.35
Transverse Poisson’s Ratio *ν*_23_	0.07	0.35

**Table 2:** Summary of predicted effective elastic properties and comparison with experimental results.

Properties	*E*_1_ (GPa)	*E*_2_ (GPa)	*G*_12_ (GPa)	*G*_23_ (GPa)	*ν*_12_	*ν*_23_
Prediction 1	137.98	8.21	4.58	3.08	0.2758	0.3243
Prediction 2	138.27	8.22	4.55	3.09	0.2747	0.3243
Prediction 3	138.49	8.25	4.55	3.08	0.2747	0.3233
Prediction 4	138.32	8.22	4.53	3.10	0.2737	0.3262
Prediction 5	138.38	8.20	4.58	3.10	0.2747	0.3262
Prediction 6	138.14	8.21	4.55	3.07	0.2758	0.3243
Prediction 7	138.33	8.24	4.59	3.10	0.2747	0.3243
Prediction 8	138.40	8.20	4.54	3.10	0.2737	0.3262
Average	138.29	8.22	4.56	3.09	0.2747	0.3249
Standard Deviation	0.152	0.016	0.019	0.011	0.00074	0.00107
Experimental [31]	138.00	11.00	5.50	3.93^*^	0.2800	0.4000
Error (%)	0.20	−25.28	−17.13	−21.391	−1.88	−18.78

* Calculated from G23=E232(1+v) .
